# Investigating individual and social behaviour of the Northern bald ibis (*Geronticus eremita*): behavioural variety and welfare

**DOI:** 10.7717/peerj.5436

**Published:** 2018-09-04

**Authors:** Caterina Spiezio, Valentina Valsecchi, Camillo Sandri, Barbara Regaiolli

**Affiliations:** 1 Research and Conservation Department, Parco Natura Viva—Garda Zoological Park, Verona, Italy; 2 Department of Comparative Biomedicine and Food Science, University of Padua, Padua, Italy; 3 Department of Animal Health Care and Management, Parco Natura Viva—Garda Zoological Park, Verona, Italy; 4 Department of Food and Agricultural Sciences, University of Bologna, Bologna, Italy

**Keywords:** Ethogram, Threskiornithidae, Welfare, Behavioural Variety Index

## Abstract

The Northern bald ibis (*Geronticus eremita*) (NBI) is one of the most threatened birds in the world. Intense conservation efforts have been undertaken and several research projects on the species are being done in Morocco and in Europe. Observing animal behaviour has been proved to be an efficient and non-invasive technique to assess the animal welfare, with the performance of a wide array of natural behaviours being one of the mostly used indicators of good mental and physical well-being. The aim of this study was to investigate the behaviour of a flock of 14 zoo-living NBI of different ages. The study focused on the variety of species-specific individual and social behaviours, in the light of reintroduction of the study juveniles in the wild. Per subject, 20 10-min. sessions were done. A continuous focal animal sampling method was used to collect individual and social behaviours. Behavioural data have been compared between adults and juveniles. Moreover, a Behavioural Variety Index (BVI) has been proposed and calculated based on previous literature describing natural ibis behaviours. The BVI might help in the evaluation of the variety of behaviours performed by each individual and the monitoring of the diversity of the behavioural repertoire of zoo animals. Our results showed that the birds performed species-specific behaviours and no abnormal behaviour was reported. Moreover, the BVI highlighted a good behavioural variety as each bird performed approximately 78% of the natural behaviours described in the Northern bald ibis and in close relative species. Our findings seem to suggest the presence of qualitative and quantitative similarities between the behavioural repertoires of the study ibises and those described in wild conspecifics, suggesting a good welfare of the colony. Finally, the BVI proposed in the current study seems to be a useful and practical tool to test behavioural diversity in zoo animals.

## Introduction

The Northern bald ibis (*Geronticus eremita*) (NBI) is listed as Critically Endangered in the IUCN Red List of species and is one of the most threatened birds in the world (Brindley et al., 1995). The situation is due to the long-lasting decline and the very small population, counting less than 250 birds in Morocco and few other individuals in relict breeding colonies in North Western Africa and in the Middle East (Boehm, 2003; Serra et al., 2004; Aourir et al., 2017). Given the precarious situation, long-term management in controlled environment becomes really important. Nowadays, European zoological gardens that are members of the EAZA (European Association of Zoos and Aquaria) actually house approximately 1150 NBI (Species360, 2016), originating from the Moroccan population, whereas another 350 birds can be found in zoos outside of Europe (Species360, 2016). Intense conservation efforts by zoos and dedicated partners have been undertaken and several research projects aiming at the reintroduction of the species are being done in Morocco and in Europe. *Proyecto Eremita*, in Spain and LIFE *Northern Bald Ibis*—*Reason for Hope in Austria* are among the most important and efficient reintroduction projects, leading to the creation of resident and migrating breeding flocks respectively. *Proyecto Eremita* aims to establish a sedentary, free-flying colony in Southern Spain through two main steps. The first step is based on hand-rearing by characterized foster parents wearing black shirts and ibis shaped helmets. In the second step, a free flying hand-reared group of ibises is established in the area and other juvenile parent-reared birds from zoos are introduced in such group. Juveniles are maintained in an aviary during a period (October –January) for acclimatisation before release. This technique facilitates the integration of the new zoo birds into the free-ranging hand-reared group (Quevedo, López & Sánchez, 2017). The study Northern bald ibises were involved in *Proyecto Eremita*, as juvenile parent-reared birds of the flock were destined for reintroduction in the wild.

The NBI is a gregarious species that lives and forages in loose flock of five to 40 individuals. Birds of the flock nest and forage together, preying upon insects, small reptiles and amphibians, small birds, small mammals and carrions (del Hoyo, Elliott & Sargatal, 1992). Wild ibis might forage for up to eight hours every day, probing the ground or the vegetation with the long bill (Cramp & Simmons, 1977; Kotrschal, 2003).

Investigating how animals cope with the environment may provide information on their mental and body stability and therefore on their welfare (Broom, 1997; Broom & Fraser, 2007). Observing animal behaviour has been proved to be an efficient and non-invasive technique to assess the animal welfare, with the performance of a wide array of natural behaviours being one of the mostly used indicators of good mental and physical health (Hill & Broom, 2009; McCormick, 2012; Hosey, Melfi & Pankhurst, 2013). The knowledge of animal behaviour and the responses to the environment performed by each individual might provide useful information to fulfil the needs of the species as well as the individuals (Hill, 2009; Hill & Broom, 2009). At the same time, the presence of abnormal behaviour might signal a compromised welfare and the incapability of the animal to cope with the environment (Garner, 2005; Hosey, Melfi & Pankhurst, 2013). Among captive birds, abnormal behaviours include species typical behaviours that are performed in excess (e.g., aggressive behaviour) or activities outside the normal behavioural pattern of the animals (e.g., self-injurious behaviour such as feather plucking) (Garner, Mason & Smith, 2003; Garner, 2005). In the NBI, abnormally sedentary lifestyle has previously been linked to serious foot problems such as bumblefoot (*pododermatitis*) (Reidarson, McBain & Burch, 1999; Samour, 2000; Tully, Lawton & Dorrestein, 2000; Vargas-Ashby & Pankhurst, 2007). Thus, investigating the behavioural repertoire and time-budget of different activities of ibises in controlled environment might be useful to detect and prevent these kind of problems.

The current study focused on a breeding flock of zoo-living NBI in Italy, involved in the *Proyecto Eremita* project. Assessing the welfare and investigating the behavioural repertoire of animals before their reintroduction in the wild seems to be particularly relevant for the success of the reintroduction. Previous studies on giraffe (*Giraffa camelopardalis*) (Veasey, Waran & Young, 1996) and mute swan (*Cygnus olor*) (Guyon, 2009) comparing the behaviour between captive and wild animals have been reporting differences in activity budgets between the two. This kind of knowledge is important to plan appropriate environmental enrichment program or enclosure design, to allow the regular performance of natural behaviour and to avoid undesirable behaviours (Veasey, Waran & Young, 1996; Guyon, 2009; Hosey, Melfi & Pankhurst, 2013). Nowadays, animal welfare studies in zoo animals have been focusing on the presence of species-specific behaviour and the absence of abnormal behaviour such as stereotypies. Comparing time budgets between wild and zoo settings seems to be a good methodology to assess welfare of the animals. However, to suggest methodologies in the assessment of good rather than acceptable welfare have become increasingly important in animal welfare science and developing indicators to address the positive welfare of each individual are very important. The variety of behaviours performed by each subject could be used as a positive welfare indicator since behavioural variety could be lost during challenging situations that could characterize controlled environments (McCormick, 2012).

The aim of this study was to investigate and describe the behaviour of a flock of 14 zoo-living NBI of different ages, focusing on the variety of species-specific individual and social behaviours performed by the flock. The analysis of the behaviour of the flock and the comparison between juvenile and adult individuals will be useful for two main reasons. On one side, it will provide information on the behavioural repertoire and activity budgets of the NBI that will be useful to assess the welfare of the zoo colony. On the other side, the behaviours performed by the study ibises might help to make informed decisions when choosing birds to be released in the wild.

To assess the positive welfare of each individual in a zoo collection is not so simple. This study aimed also to provide a Behavioural Variety Index that could be used as a tool to routinely monitor the zoo animals’ positive welfare. Spot checks are becoming a popular method to monitor hygiene behaviours and the incidence of diseases in human society (Webb et al., 2006). In the current research, basing on data collected through continuous recording of focal animal behaviour, a method used in the human literature to assess hygiene behaviours, was reviewed to propose the so called Behavioural Variety Index (BVI). The BVI might help in the evaluation of the variety of behaviours performed by each individual and monitor the diversity of the behavioural repertoire of zoo animals. The comparison of the data on behavioural time budgets between the zoo individuals and wild conspecifics as well as the BVI should provide quantitative and qualitative information on the animal welfare state.

## Materials & Methods

### Study subjects & area

This study was carried out in 2013 with 14 NBI housed in a breeding flock at Parco Natura Viva, an Italian zoological garden. The flock was made of 10 males and four females of different age. In particular, the seven ibises hatched between 2012 and 2013 were considered juveniles, whereas the other subjects were considered adults, as they were sexually mature and had the morphological features of adult NBI (del Hoyo, Elliott & Sargatal, 1992; Sinclair & Ryan, 2010) (Table 1). The approximate average age (± standard error) of the ibises was 106.86 ± 5.77 months for adults and 7.43 ± 2.21 months for juveniles (average age of the whole flock: 57.14 ± 14.10). All subjects were parent-reared. Birds were identified through a band on one leg. The bands differed in colour and letters (two-letter combination) (Table 1).

**Table 1:** Northern bald ibises of the study. For each subject, the table reports the identification band (ID-Band), the sex (M  = male, F  = female), the year of birth (Born in) and the age class.

ID-band	Sex	Born in	Age class
YK-blue	M	2003	Adult
CW-red	F	2003	Adult
YV-red	F	2004	Adult
XP-blue	M	2004	Adult
FA-red	M	2005	Adult
CN-green	F	2006	Adult
YZ-green	F	2006	Adult
UA-red	M	2012	Juvenile
RK-red	M	2012	Juvenile
TY-green	M	2013	Juvenile
HZ-green	M	2013	Juvenile
GA-green	M	2013	Juvenile
TV-green	M	2013	Juvenile
CX-green	M	2013	Juvenile

The ibises were housed in a mixed-species enclosure, along with a male griffon vulture (*Gyps fulvus*). The enclosure was a 169 m^2^ aviary with a perimeter of 51.44 m and a height of approximately 7 m. The aviary size and the presence of perches allowed the birds to glide and partially to slap from one side of the aviary to the other one. The aviary was provided with trees and bushes, rocks, wooden logs, trunks, a small running-water pond, two wooden houses serving as a shelter from weather conditions and several wooden nest boxes in the top part of the area. Different perching branches were provided throughout the aviary. At the front of the aviary, four small viewing windows allowed the view of the ibises to the public, reducing the impact of visitor presence on the bird. Food was provided daily to the ibis in the middle of the enclosure, around the pond. The ibis diet was made of minced meat, chicks, insects (mainly mealworms) and fish. The food was presented either in bowls (meat, chicks and fish) or scattered around in the enclosure as foraging enrichment (mealworms). Food was provided to the ibises once a day, generally in the late morning.

### Procedure and data collection

The study was carried out over October and November, outside the breeding season of the ibises, starting in mid-February (del Hoyo, Elliott & Sargatal, 1992). Per each subject, a total of 20 10-minute sessions were done (12,000 s of observation per subject). In particular, two sessions per day were carried out, one in the morning (10.30 AM–1.30 PM) and one in the afternoon (2.30 PM–5.30 PM). Over the study sessions, the ibises were observed in a random sequence, to collect the behaviour of each subject over the whole observation period, from the beginning (10.30 AM and 2.30 PM) to the end of the data collection time, both in the morning and in the afternoon.

**Table 2:** Behavioural ethogram of the study. The behaviour list has been prepared based on preliminary observation of the flock and on previous studies on the Northern bald ibis as well as on relative species (Hirsch, 1979; Kopij, 1998; Pegoraro & Föger, 2001; Mark, 2007; Vargas-Ashby & Pankhurst, 2007; Clark et al., 2012; Fernández-Juricic, 2012; Moulton et al., 2013; Frigerio et al., 2016).

**Individual behaviour**		
	Alertness	Moving the head, stretching the neck listening or watching something in the surrounding.
	Attentive behaviour	Being vigilant, scanning the environment.
	Comfort behaviour	Tidying and cleaning the own feathers with the bill, scratching, bathing and sunbathing (fanning the wings to warm up in the sun).
	Exploration	Looking for food probing the ground, tree branches, crevices and other elements of the aviary with the bill.
	Flight	Flying or hopping from one point to the other of the enclosure.
	Walking	Walking around the aviary.
	Maintenance	Eating the food provided in the feeding point, defecating, drinking.
	Manipulation/play	Manipulating food, objects, twigs, rocks or other inanimate items found in the aviary using the bill or the legs.
	Resting	Standing on one or both legs, with the head turned back and tucked beneath the wings.
**Social behaviour**		
	***Social affiliative behaviour***	
	Preening	Tidying and cleaning the feathers of a conspecific with the bill.
	Other affiliative	Greeting displays including head tossing, head rubbing, mutual bill shaking; observing conspecifics.
	***Social agonistic behaviour***	
	Aggression	Pecking towards approaching birds, hitting a conspecific with the bill or with the legs.
	Agonistic display	Bill gaping, ruffling the helmsman feathers, moving or lunging toward conspecifics, touching slightly with the tip of the bill. Anti-predatory behaviour (e.g.: mobbing).
**Not visible**		
	Out of sight	The ibis spends time “out of sight” and it is not possible to see what the individual is doing. In particular, the bird is in sheltered areas such as nest boxes, rock crevices or behind trees and bushes.

A continuous focal animal sampling method was used to collect durations of individual and social behaviours (Altmann, 1974) performed by the birds (Table 2). We also recorded the time ibises spent out of sight, as if performed in excess this could be informative on the animal state. Indeed, being out of sight is particularly relevant in a zoo environment, as wild animals can sometimes try to hide in the presence of visitors, whenever they are perceived as negative stimuli (Carlstead, Brown & Strawn, 1993; Sellinger & Ha, 2005; Davey, 2006; Morgan & Tromborg, 2007; Hosey, Melfi & Pankhurst, 2013; Spiezio et al., 2017). For this reason, recording the time spent out of sight has become important and exhaustive to assess the effect of zoo visitors on the behaviour and welfare of the animals (Farrand, 2007). In the current study, when out of sight the focal ibis was not visible to the observer from the visitor viewing window because the bird was in sheltered areas such as nest boxes, rock crevices or behind trees and bushes. The behavioural ethogram of the study is reported in Table 2 and was prepared basing on preliminary observation of the flock and on previous studies on the Northern bald ibis as well as on relative species (Hirsch, 1979; Kopij, 1998; Pegoraro & Föger, 2001; Mark, 2007; Vargas-Ashby & Pankhurst, 2007; Clark et al., 2012; Fernández-Juricic, 2012; Moulton et al., 2013; Frigerio et al., 2016).

The study was carried out through the live observation of the birds, using non-invasive or stressful techniques. The study procedure was in accordance with the EU Directive 2010/63/EU and the Italian legislative decree 26/2014 for Animal Research. All procedures performed in the study were in accordance with the ethical standards of Parco Natura Viva as the research was approved by Parco Natura Viva ethical committee and by the local veterinary authority.

### Behavioural variety index

To investigate the behavioural variety in the study flock, we revisited the method proposed by Webb and colleagues (2006) to measure hygiene behaviours in humans. These authors grouped different behaviours in four groups (water, food, personal and household hygiene) and calculated a Hygiene Behaviour Index for each group. Basing on all these indices, a Summary Hygiene Index was created. Following this methodology, after having examined the existing literature, we prepared a list of natural behaviours collected by previous researchers on the NBI as well as on close relative species, in both wild and captive settings (Hirsch, 1979; Kopij, 1998; Pegoraro & Föger, 2001; Mark, 2007; Vargas-Ashby & Pankhurst, 2007; Clark et al., 2012; Fernández-Juricic, 2012; Moulton et al., 2013; Frigerio et al., 2016). The list included 18 items that were grouped in four subsets basing on the behavioural function (Table 3) (Kopij, 1998). Comfort and Social behaviours described as typical of the breeding season and the incubation period (e.g., copulation, nest building) were discarded, as the current study was done in the non-breeding period. Four specific indices were related to the resulting groups: Routine behaviour (R; score: 0–5), Exploration/Attention behaviour (E; score: 0–5), Comfort behaviour (C; score: 0–3) and Social behaviour (S; score: 0–5). These indices were used to create the Behavioural Variety Index (BVI). When calculating the indices for the study ibises, firstly each item was scored as 0 or 1, with 1 representing the performance of the behaviour. Then, each index was calculated as the sum of the behavioural items performed by each ibis and the indices’ score ranged from 0 to the total number of behavioural items found for each group (Table 3). Each index was calculated for each individual. A BVI was calculated for each subject and resulted from the sum of the four indices. Basing on previous literature on species behavioural ethogram, the BVI might be useful to measure the variety of positive behaviours of the animals in a controlled environment, representing a tool to assess their welfare and needs (McCormick, 2012).

**Table 3:** Behaviours of the Northern bald ibis described in previous research. Behaviours have been grouped in four main groups and the number of behaviours per group was used to develop indices of the ibis behavioural variety in the study flock. The possible score for each group is calculated starting from the number of behaviours that have been previously described in the Northern bald ibis and in close relative species (*Geronticus calvus*).

Routine b. (B)	Exploration/Attention b. (E)	Comfort b. (C)	Social b. (S)
Flight^a^^,^^g^	Probing ground^a^^,^^e^^,^^g^	Preening & scratching^a^^,^^g^	Agonistic display^a^^,^^d^
Walking^g^	Probing vegetation^g^	Bathing^a^	Aggression^a^^,^^e^^,^^g^
Resting^a^	Manipulation/Play^b^	Sun bathing^a^	Preening^a^^,^^b^^,^^c^^,^^e^^,^^f^
Defecation^a^	Attentive behaviour^i^		Greeting (sweeping head movements)^a^^,^^b^^,^^c^^,^^d^^,^^e^
Eating (food from zookeeper)/drinking^g^	Alertness^h^		Anti-predatory (mobbing)^a^
**Possible score: 0–5**	**Possible score: 0–5**	**Possible score: 0–3**	**Possible score: 0–5**

**Notes.**

a Kopij (1998).

b Mark (2007).

c Clark et al. (2012).

d Pegoraro & Föger (2001).

e Hirsch (1979).

f Frigerio et al. (2016).

g Vargas-Ashby & Pankhurst (2007).

h Moulton et al. (2013).

i Fernández-Juricic (2012).

### Statistical analysis

According to Kolmogorov–Smirnov goodness of fit tests, not all data were found to be normally distributed (out of sight: *p* = 0.031; Overall affiliative behaviour: *p* = 0.018; Flight: *p* = 0.011, preening and aggression: *p* < 0.01; for all other behavioural categories: *p* > 0.05). Therefore, data were analysed using non-parametric statistic tests with significance level set at *p* < 0.05 (Siegel & Castellan, 1992). In particular, to compare differences in the duration of individual and social behaviours between adult and juvenile ibises, a Mann–Whitney test was used. The same test was used to compare the BVI between age-groups. Moreover, Spearman correlations were run to measure the relationship between each index and the BVI. Bonferroni adjustment was used to correct *p*-values in the presence of multiple comparisons. All tests were two-tailed. In the results, the reported percentages were calculated on the total observation time (seconds) of the flock (280 sessions, 168,000 s).

## Results

**Figure 1:**
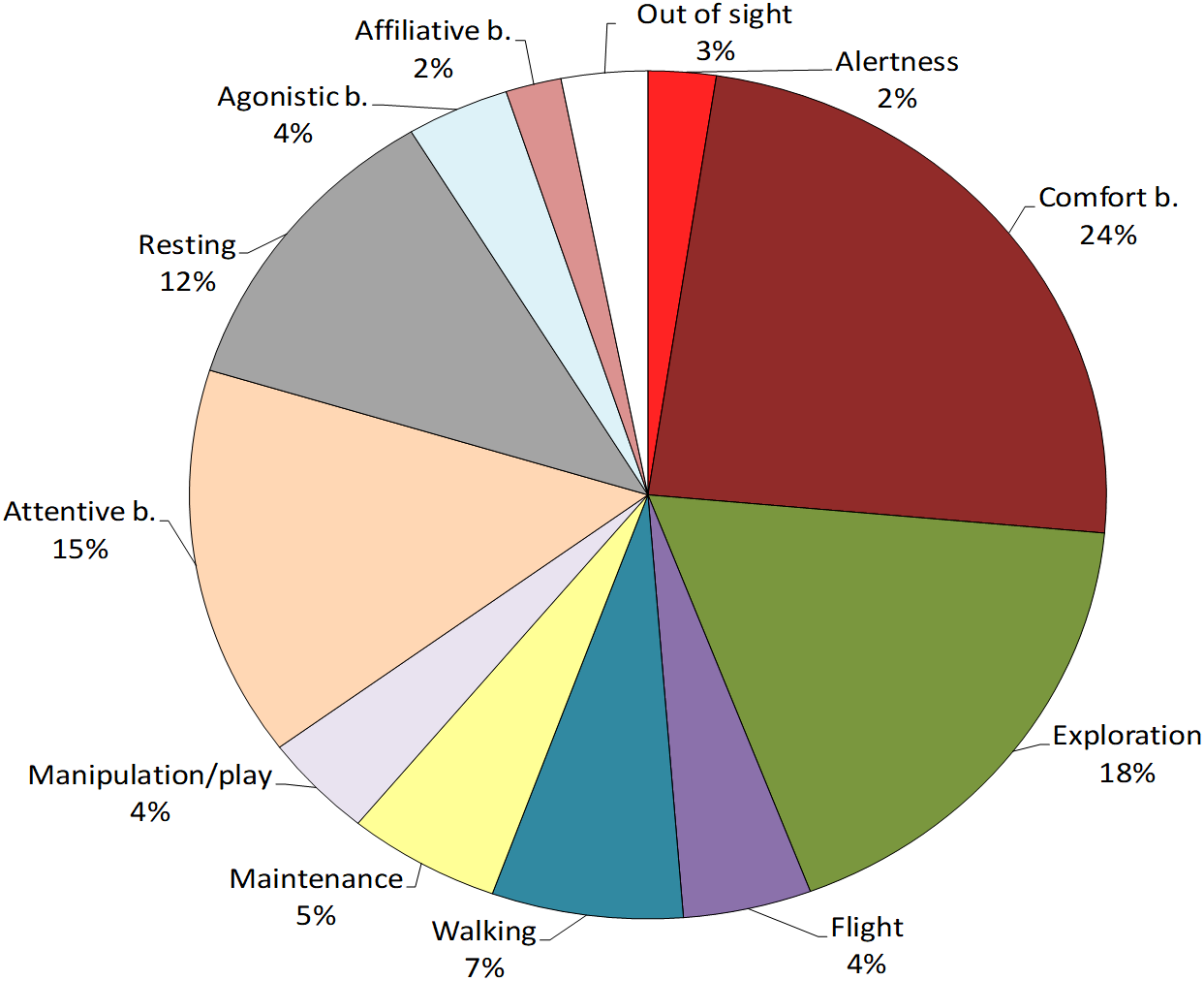
Behavioural repertoire of the study Northern bald ibis. The pie-chart reports the % total duration (seconds) of individual and social behaviours performed by the study flock. Percentages are calculated on the total observation time of the colony (20 sessions, 168,000 s).

First, the most performed behavioural class was individual behaviour (91%), followed by social behaviour (6%), and out of sight (3%) (Fig. 1). No abnormal behaviour was reported. To verify the presence of age-related differences in the ibis behaviour, we compared the time spent in each class between juveniles and adults. Mann–Whitney tests revealed that juveniles performed significantly more individual behaviour than adults (*U* = 7, *p* = 0.03) whereas no differences were found for social behaviour (*U* = 23, *p* = 0.897) and out of sight (*U* = 19, *p* = 0.522).

### Performance of individual behaviours

**Figure 2:**
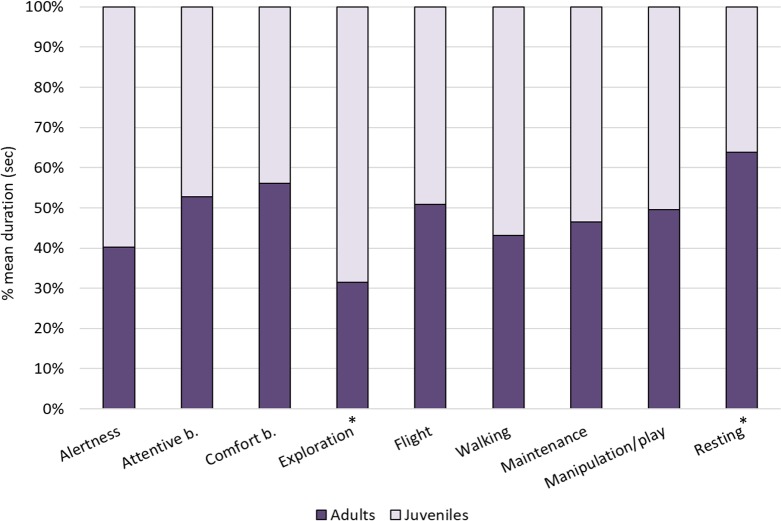
Individual behaviours in adults and juveniles. The bar-chart reports the % mean duration (in seconds) of different individual behaviours performed by both adult and juvenile ibises. Asterisks indicate categories in which a significant difference between age groups was found (Mann–Whitney test: *p* < 0.05).

Within individual behaviours, the most performed behavioural category was comfort behaviour (24%), followed by exploration (18%), attentive behaviour (15%), resting (12%) and walking (7%), whereas other categories were performed for less than 5% of the total time (see Fig. 1). When comparing the behaviours between age-groups, Mann–Whitney tests revealed significant differences in exploration, which was performed more by juveniles than adults (*U* = 0, *p* = 0.002) and resting, which was performed more by adults than juveniles (*U* = 5, *p* = 0.015). No other differences were reported (*p* > 0.05, see Table 4 for median, interquartile range, *U* and *p* values) (Fig. 2).

**Table 4:** Individual and social behaviours in adult and juvenile Northern bald ibises, Median (IQR) per behavioural category for adults and juveniles. Below the data of the two age-groups the *U* and *p* from the Mann–Whitney test (MW) are also reported.

		*Alertness*	*Attentive b.*	*Comfort b.*	Exploration^*^	
Individual b.	*Adults*	232 (157–345)	1,893 (1,318–2,103)	3,225 (2,869–4,384)	1,097 (992–1,868)	
	*Juveniles*	339 (206–482)	1,741 (1,511–1,951)	2,519 (2,121–2,711)	2,834 (2,485–3,087)	
	*MW*	*U* = 15; *p* = 0.250	*U* = 20; *p* = 0.610	*U* = 11; *p* = 0.097	*U* = 0; *p* = 0.002	
		*Flight*	*Locomotion*	*Maintenance*	*Manipulation*	Resting^*^
	*Adults*	517 (320–560)	699 (493–786)	652 (445–710)	422 (247–598)	1,540 (1,440–2,145)
	*Juveniles*	510 (478–690)	1,010 (546–1,144)	720 (551–834)	589 (298–633)	1,015 (596–1,425)
	*MW*	*U* = 23; *p* = 0.897	*U* = 15; *p* = 0.250	*U* = 15; *p* = 0.250	*U* = 21; *p* = 0.704	*U* = 5; *p* = 0.015
		*Aggression*	*Agonistic display*	*Other affiliative*	*Preening*	* *
Social b.	*Adults*	29 (0–151)	274 (242–366)	217 (76–250)	81 (0–248)	
	*Juveniles*	25 (23–131)	480 (355–539)	132 (89–170)	0 (0–0)	
	*MW*	*U* = 23.5; *p* = 0.952	*U* = 10.5; *p* = 0.085	*U* = 15; *p* = 0.250	*U* = 10.5; *p* = 0.085	

**Notes.**

*Indicate behavioural categories for which a significant difference between adults and juveniles was found (*p* < 0.05).

### Performance of social behaviours

Within social behaviours, affiliative and agonistic behaviours were performed for 2% and 4% of the total time respectively (Fig. 1). More specifically, for affiliative behaviours, we considered preening and other affiliative behaviours, including greeting displays such as head tosses, head rubbing and mutual bill shaking (see Table 2). The study ibis flock spent 0.8% of the time preening and 1.2% doing other affiliative behaviours. For agonistic behaviours, we considered aggression and agonistic display, including slight aggressive interactions such as dominance displays and displacement. The ibis flock performed agonistic display for approximately 3% and aggression for 0.6% of the total time.

**Figure 3:**
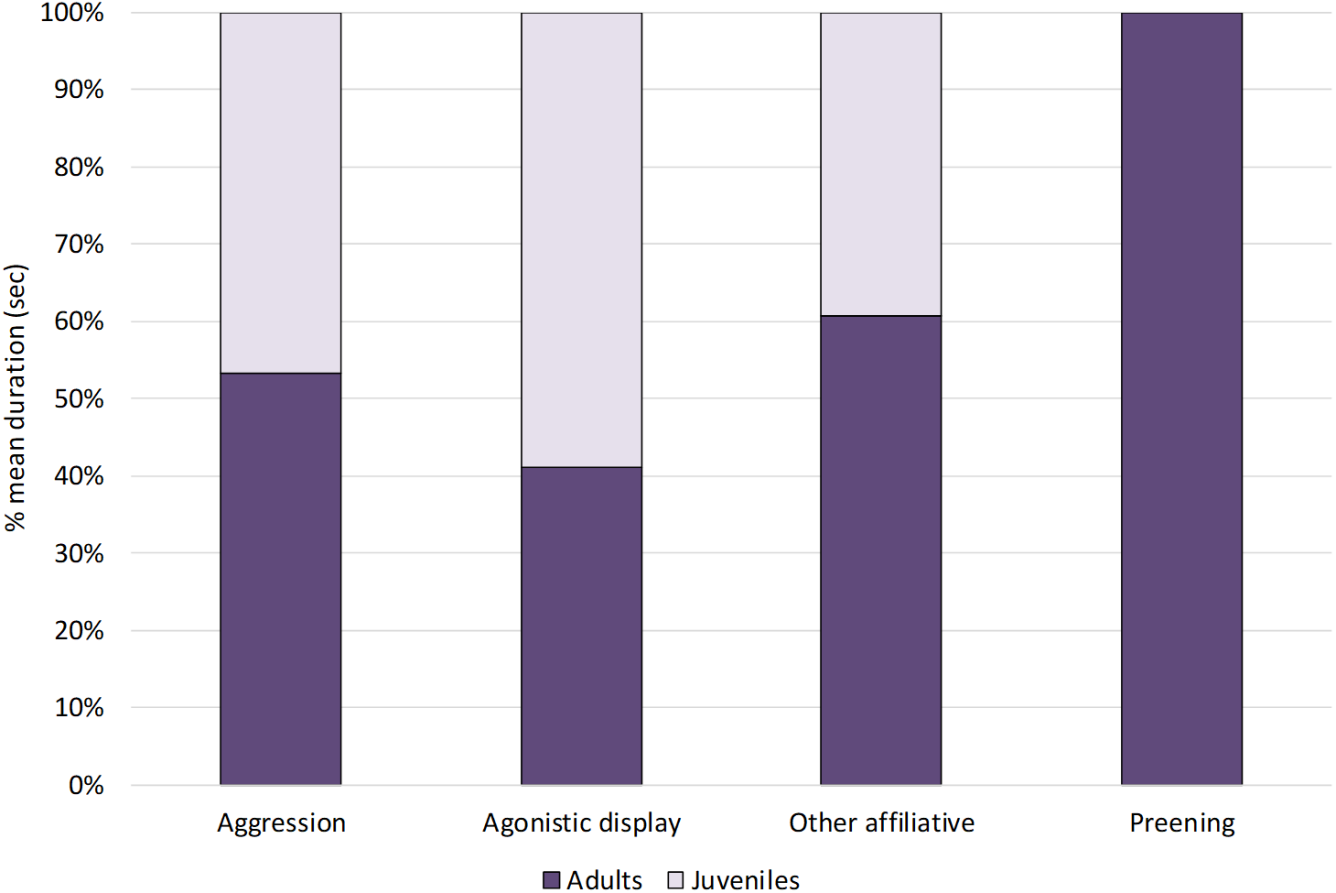
Social behaviours in adults and juveniles. The bar-chart reports the % mean duration of different social behaviours performed by both adult and juvenile ibises.

When comparing the behaviours between age-groups (Fig. 3), Mann–Whitney tests revealed no significant differences between juveniles and adults (*p* > 0.05, see Table 4 for median, interquartile range, *U* and *p* values).

**Figure 4:**
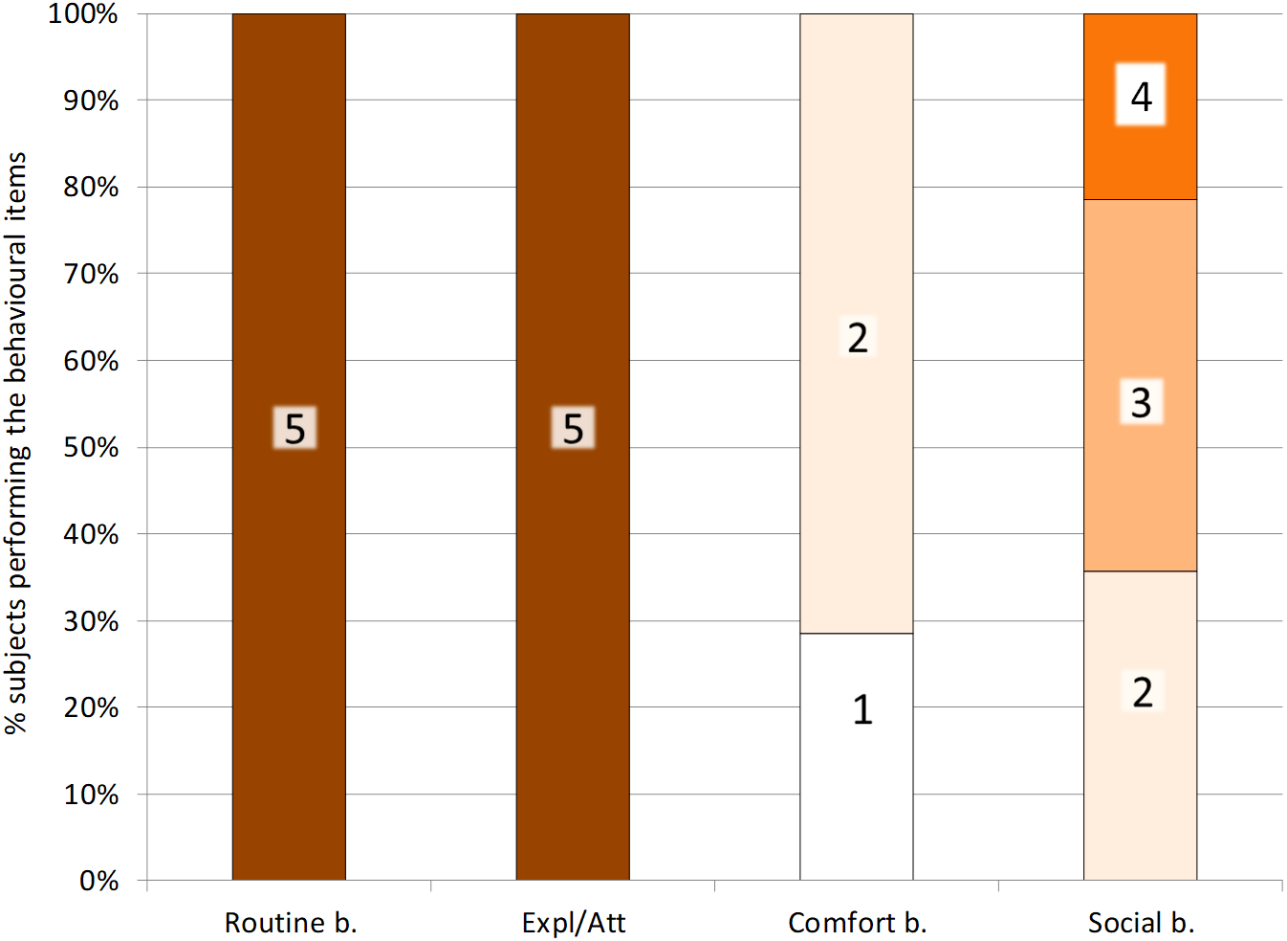
Distribution of scores for the four behavioural indices. Different bar boxes report the number of behavioural items that have been performed per group of behaviours: Routine behaviour, Exploration/Attention behaviour (Expl/Att), Comfort behaviour, Social behaviour. The total number of behaviours (score) that could be performed varied from 3 (Comfort b.) to 5 and has been specified in each box of the plot.

**Figure 5:**
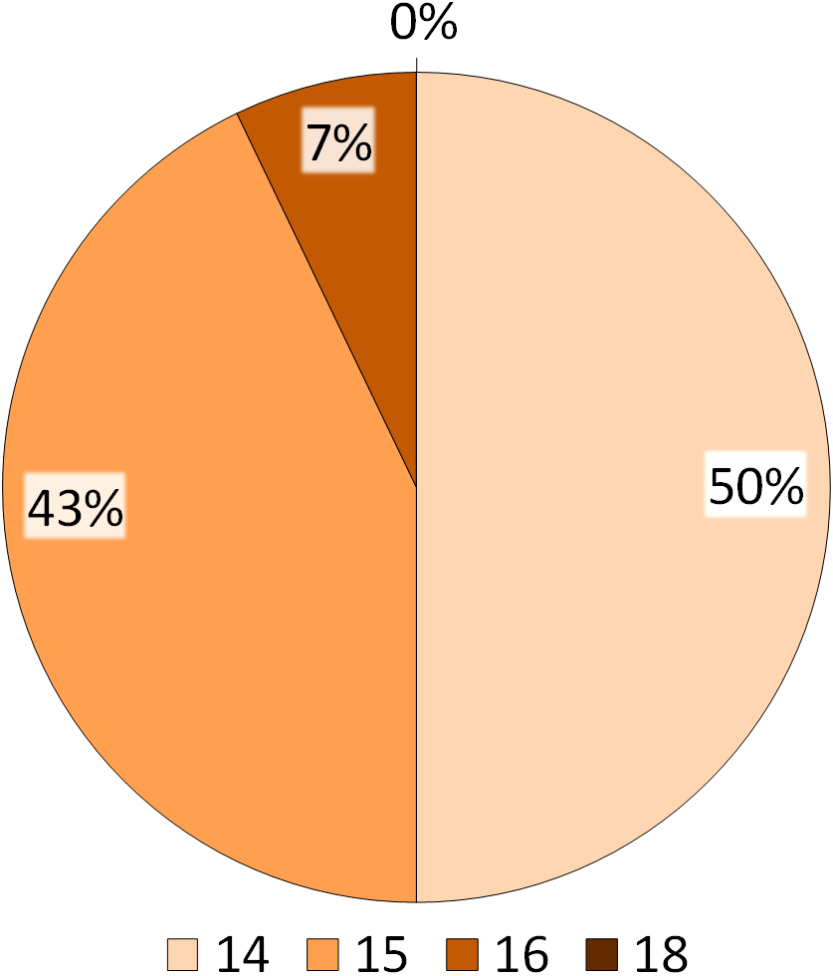
Summary of the BVI scores. The numbers indicated in the legend refer to the total number of behavioural items performed by the ibises, whereas the percentages reported on the pie refer to the number of subjects performing the behavioural items.

### Behavioural variety

The distribution of the 18 behavioural items reported in the NBI and the four corresponding indices are presented in Figs. 4 and 5. Figure 4 shows that all subjects (100%) had the maximum index for Routine behaviour (*R* = 5) and for Exploration/Attention behaviour (*E* = 5). For Comfort behaviour, all items but Bathing (2/3) were found, with approximately 30% and 70% of the ibises having a score of 1 and 2 respectively (C ≤ 2). For Social, 36% of the subjects had a score of 2 (*S* = 2), 43% had a score of 3 (*S* = 3) and 21% had a score of 4 (*S* = 4) (Fig. 4). As reported in Fig. 5, in summary, the BVI scores revealed that all ibises had a score ≥14. In particular, seven subjects (50%) had a BVI of 14, six subjects (43%) had a BVI of 15 and one subject (7%) had a BVI of 16 (Fig. 5). No significant difference in the BVI scores between adults and juveniles was found (*U* = 19.5, *p* = 0.569). We ran Spearman correlations with Bonferroni adjusted *p*-value of 0.025 (0.05/2) to assess the relationship between each index and the BVI. A significant positive correlation was found between Social behaviour and the BVI (*Rho* = 0.803, *p* = 0.001) but no correlation was reported between Comfort behaviour and BVI (*Rho* = 0.044, *p* = 0.882). As the R and E indices had a standard deviation of 0, Spearman correlations could not be carried out with these distributions and the BVI.

## Discussion

This study highlighted that NBI showed a species-specific behavioural repertoire and no abnormal behaviour was reported. The most performed behavioural category was comfort behaviour, including self-preening and scratching, which was performed for 24% of the total time. Very similar time budgets have been reported in other studies on this species in controlled environment (Vargas-Ashby & Pankhurst, 2007) and on wild non-breeding subjects of Southern bald ibis (Kopij, 1998). In the wild, the rate of comfort behaviours has been found to be high in both the Northern (Wackernagel, 1964) and Southern bald ibis (Kopij, 1998). These species have colonial habits and are therefore more likely to be infested by ectoparasites (Brown & Brown, 2004; Fecchio et al., 2011; Rifkin, Nunn & Garamszegi, 2012). Preening and other comfort behaviours such as scratching, bathing and sun bathing have been described as strategies used by birds to deter ectoparasites and release local irritation caused by them (Clayton & Cotgreave, 1994; Clayton et al., 2010). Thus, comfort behaviour reported in the current study seems to agree with previous findings on this species as well as on other birds, confirming the importance of this category for these species.

The second most performed behavioural category was exploration, intended as looking for food by probing the ground and the vegetation of the aviary with the bill. This behaviour is particularly relevant as wild ibises might forage for six to eight hours a day (Cramp & Simmons, 1977; Kotrschal, 2003). Despite the study birds are fed daily in a specific area of the aviary, the enclosure design with the presence of rocks, grass and bushes seems to be enriching for the birds, stimulating the performance of natural feeding behaviour and enhancing their welfare (Vargas-Ashby & Pankhurst, 2007).

The third most performed behaviour was resting, as ibises spent 12% of the time standing with the head turned back and the bill beneath the wings. Inactive behaviours are quite common in captive bare environments and have been previously hypothesized to contribute to feet problems (e.g., bumblefoot) in birds (Samour, 2000; Tully, Lawton & Dorrestein, 2000). However, the study ibises rested for a similar amount of time as reported in wild Southern bald ibis, in which non-breeding birds have been found to sleep for approximately 10% of the time (Kopij, 1998).

Among other individual behaviours performed with a relatively low percentage, we found that all ibises showed manipulation, interacting with inanimate objects such as twigs and leaves. This behaviour has been previously described as object play in the NBI and has been proposed as a good indicator of stress reduction and well-being (Fagan, 1981; Hall, 1998; Mark, 2007). Thus, the flock seems to be managed appropriately with low levels of stress and a good physical and emotional well-being (Fagan, 1981; Mark, 2007).

NBI are gregarious birds living in large flocks and interactions with conspecifics are complex and diverse (Hirsch, 1979; Kopij, 1998). Regarding social behaviour, affiliative behaviours were preening and other affiliative behaviours, intended as greeting displays that are used by the ibises during both the breeding and non-breeding seasons (Hirsch, 1979; Kopij, 1998). Outside the breeding seasons, greetings by sweeping head movements are important during contact with pair, family and group members, functioning as appeasement signals and social cohesion (Pegoraro & Thaler, 1985; Kopij, 1998; Pegoraro & Föger, 2001). Affiliative behaviour of the study flock encompassed mainly greeting displays, whereas preening was performed by a few subjects and for a reduced amount of time (0.8%). This finding might be due to the fact that preening is often associated with greeting displays that are more common in the breeding season (Hirsch, 1979; Kopij, 1998; Clark et al., 2012), whereas this study took place after this period. Within agonistic behaviours, threats and slightly aggressive interactions (agonistic display) were the most common behavioural categories (3%), whereas aggression intended as hitting conspecifics was performed with a lower rate (0.6%). In the wild, aggression is generally infrequent in ibises (del Hoyo, Elliott & Sargatal, 1992; Kopij, 1998) as birds prefer to discourage or displace other individuals by simply touching them with the tip of the bill or performing threat displays rather than a fight (Hirsch, 1979; Kopij, 1998). Therefore, social behavioural patterns of the study ibises seem to resemble those reported in their wild conspecifics.

“Out of sight” has been found to be informative of the animal welfare state as it could represent a choice because animals seek out and use areas in which they are not visible to the visitors (Carlstead, Brown & Strawn, 1993; Sellinger & Ha, 2005; Davey, 2006; Morgan & Tromborg, 2007; Hosey, Melfi & Pankhurst, 2013; Spiezio et al., 2017). Providing animals with privacy is important and needs to be considered for appropriate enclosure design (Hosey, Melfi & Pankhurst, 2013). The study found that Northern bald ibises spent a low amount of the observation time out of sight (3%) in comparison with other individual and social behaviours. Together with the lack of abnormal behaviour, these findings may suggest that the welfare of the flock seems not to be compromised.

Regarding the comparison of behaviours between age-groups, the behavioural repertoire of adults and juveniles seemed to be similar. Overall, juvenile ibises performed significantly more individual behaviours than adults. In particular, exploration was shown more by juveniles than adults, whereas the opposite pattern was found for resting. Previous studies on European blackbirds (*Turdus merula*) (Desrochers, 1992), nuthatches (*Sitta europea*) (Enoksson, 1988) and Herring gulls (*Larus argentatus*) (Cristol et al., 2017) showed that juvenile birds are generally less efficient foragers than adults, probably due to inexperience in prey/food detection and handling or competition with adults. Therefore, it is possible that juvenile ibises of this study needed to spend more time looking for food for two reasons: (1) dominant adults tend to monopolise the food provided by the zookeepers, forcing juveniles to look for food for longer time and throughout the aviary; (2) the reduced efficiency of juveniles in finding and catching preys in the enclosure led to a greater amount of time spent in this activity. However, more studies on greater samples of ibises are needed to investigate feeding behaviour in juveniles and adults, focusing on the time spent in this activity as well as on the performance in finding and processing food items. Despite social groups imposing constraints on subordinate immature individuals, juvenile birds gain from social learning, observing and practicing adults’ behaviours in social and foraging contexts (Griffin, 2004; Skòrka, Lenda & Sutherland, 2016). The lack of important differences between age-groups in the current study suggest that juvenile ibises behaved similarly to adults and may have learned most of the necessary behavioural skills typical of the species. Thus, juvenile birds seem to be ready for the release into wild flocks of Northern bald ibises.

The variety of natural behaviours shown by captive animals has been hypothesized as a possible indicator of the animal physical and mental health (Broom, 1997; Hill & Broom, 2009). To evaluate the welfare of the study NBI, we developed and tested a Behavioural Variety Index (BVI), by revisiting the Hygiene Index described in the study of Webb et al. (2006). The BVI allowed the comparison of the behavioural repertoire of our sample of NBI with natural behaviours that have been previously described in this bird and in close relative species. Basing on our results, 16 out of 18 natural behaviours described for the NBI have been found in the study flock. Overall, each ibis had a BVI equal or greater than 14, meaning that each bird performed approximately 78% of the behavioural items reported in the list (Table 3). In particular, 43% of the ibises had a BVI of 15, performing approximately 83% of the behavioural items in the list and 7% of the ibises had a BVI of 16, performing approximately 90% of the behavioural items. Among the behaviours included in the list, the two items that were not reported in the study ibises were anti-predatory behaviour (within S) and Bathing (C). The absence of anti-predatory activities such as mobbing might be due to the lack of important threats and predators in the zoo setting. However, despite the study ibises seemed not to react to predators as reported in the wild, they showed alertness (2%) and were watchful and ready to respond in the case of threatening situations. Moreover, the entire flock performed all the comfort behaviours that have been previously described for the NBI, except for bathing. The study ibises had ad libitum access to a pond in the aviary and it is possible that they did not display bathing due to the weather and climate conditions, as the study was carried out in autumn. Moreover, bathing has been rarely reported even in wild ibises in specific periods of the years (Kopij, 1998). Taken together, these findings seem to suggest that the behaviour of the study ibises is similar to that reported in the wild and a good behavioural variety can be hypothesized, as all subjects performed approximately 78% of the natural behaviours that have been previously described in ibis species. As for most of the behavioural time budgets, no significant differences between adults and juveniles in behavioural variety were found.

## Conclusion

Our findings seem to highlight the presence of qualitative and quantitative similarities between the behavioural repertoires of the study NBI and that described in wild conspecifics. Together with the absence of abnormal behaviour, this suggests a good welfare of the colony, even if other parameters need to be evaluated (e.g., physiological parameters). Moreover, as the behavioural differences between age groups were very few and less than similarities, the juvenile ibises were considered suitable and ready for reintroduction in the wild. Preparing detailed ethograms of zoo animals might be helpful in the conservation of endangered species by providing zoo staffs and conservationists with a baseline of species behaviour (Sutherland, 1998; Smith & Wassmer, 2016). These ethograms can help to understand and fulfil the animal behavioural needs as well as to improve our knowledge on how a species interacts with conspecifics, heterospecifics, humans, and the environment, improving effective in-situ and ex-situ conservation (Lichtenberg & Hallager, 2008; Gokula, 2011; Smith & Wassmer, 2016). However, given that the group of birds considered in the current study is relatively small and sexually biased, future research is needed to obtain more reliable/representative information on this species in zoos. Finally, the BVI proposed in the current study seems to be a useful and practical tool to test behavioural variety between zoo-living animals and their wild counterparts or more in general between two distinct groups of animals living in different environments. Thus, the BVI seems to be a relevant methodology when measuring animal welfare in terms of performance of positive behaviours, basing on the species behavioural repertoire that has previously been described in both wild and captive settings.

**Note:** In 2016, the seven juveniles NBI involved in the current study have been released in the wild as part of the *Proyecto Eremita*. The ibises are now living in a free-ranging flock in Spain (Sierra del Retín de la Janda), where they joined an existing group of NBI and started breeding. Data are published now, after three years of birds in the wild.

##  Supplemental Information

10.7717/peerj.5436/supp-1 Supplemental Information 1 Ibis behaviour raw data Durations of individual and social behaviours per subject. The BVI sheet reports the number of behaviuoral items performed by each subject.

## Additional Information and Declarations

### Data Availability

The following information was supplied regarding data availability: The raw data are provided in Supplemental Information 1.
